# Supplementary material for: Patient-Relevant Costs for Organ Preservation versus Radical Resection in Locally Advanced Rectal Cancer
Source: Cancers (Basel). 2024 Mar 26;16(7):1281. doi: 10.3390/cancers16071281 (PMC11011197; doi:10.3390/cancers16071281)

Patient-relevant costs for organ preservation versus radical resection in locally advanced rectal cancer

Supplementary Material

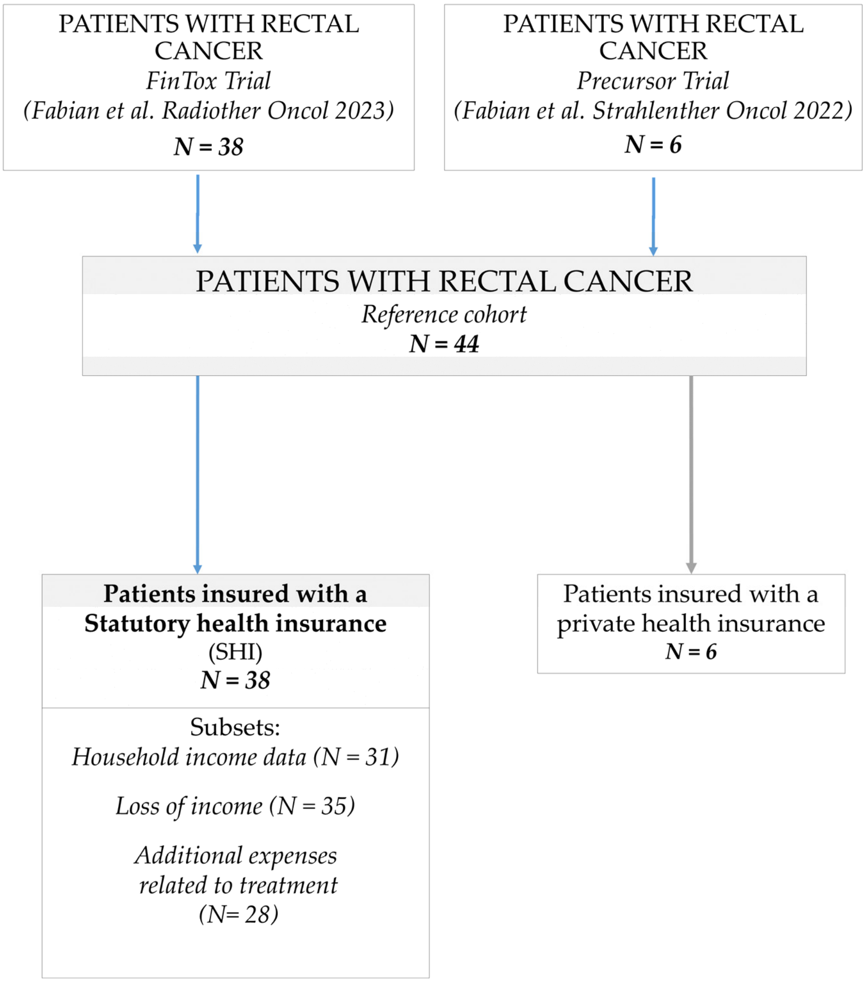

Figure S1 A flowchart displaying the composition of reference cohort from the “FinTox”-Trial and its precursor trial. Patients insured with private health insurances (N = 6) were not included in the final analysis.

Table S1: Overview of costs per state for Non-operative Management (NOM, I). Relevant data for income calculation was retrieved from Table 3 and Table 4. Costs for follow-up after local recurrence in this group (NOM, I) were assumed to be equal to costs for follow-up after resection in Group II (CRT + resection). § Co-payment expenses were cropped at the general limit of 822 € / month (primary treatment) or 411 € /month (recurrent disease). Two one-way drives were calculated per treatment. Follow-up examination schedule was time-dependent according to current guidelines (see main article). Imaging visits during follow-up might be combined so that the number of drives does not total the number of examinations. Driving expenses were calculated from patient-relevant co-payment and costs for non-refunded drives. *Maximum co-payment for hospitalization: 280 € / year (28 nights). $ Loss of salary was calculated from the difference between gross income and the general limit for sickness pay (70% of gross income) after 6 weeks of absence from work. In case of combined failure, no resumption of work was assumed and the difference between average income and disability pension was calculated according to Table 4. † No total number calculated – ongoing systemic / palliative treatment after diagnosis of recurrence, no co-payment necessary (exceeding co-payment threshold).

**NOM (Group I)**	**Transition state /**	**Stable disease**		**Recurrence**		
**Costs per year**	**Primary treatment**	**Follow-up<br>Year 1**	**Follow-up<br>Year 2 - 5**	**Local**	**Distant**	**Local and distant**
Total number of drives for treatments<br>*Correlating treatment and number of drives (one-way)*	98<br>Visits for primary diagnosis: 4<br>Treatment preparation: 4<br>Radiotherapy: 56<br>Interim staging: 2<br>Consolidative chemotherapy: 32	12<br>3x Rectoscopy + clinical examination, 3x MRI, 2x CT chest / abdomen: 2	8<br>2x Rectoscopy + clinical examination, 2x MRI, 1x CT chest / abdomen: 2	10<br>Visits for diagnostics / follow-up: 8<br>Resection: 2	10<br>Visits for diagnostics / follow-up: 8<br>Resection: 2	†<br>Visits for diagnostics / staging: 4
Co-payment for transportation [€]§<br>*Correlating treatment and number of drives (one-way)*	40 € (8)<br>Visits for primary diagnosis: 2<br>Treatment preparation: 2<br>Radiotherapy: 2<br>Consolidative chemotherapy: 2	0	0	10 € (2)<br>Resection: 2	10 € (2)<br>Resection: 2	n.a. §
Transportation costs, non-refunded [€]<br>*Correlating treatment and number of drives (one-way)*	31.56 € (6)<br>Visits for primary diagnosis: 2<br>Treatment preparation: 2<br>Staging: 2	63.12 (12)<br>Follow-up visits (see above)	42.08 (8)<br>Follow-up visits (see above)	42.08 €<br>Visits for diagnostics / staging: 4<br>Follow-up visits: 4	42.08 €<br>Visits for diagnostics / staging: 4<br>Follow-up visits: 4	21.04 € (4)<br>Visits for diagnostics / staging: 4
Co-payment for medication [€], number of prescriptions§	100 € (10)	0	0	20 € (2)	20 € (2)	§
Co-payment for hospitalization for resection (number of nights)*§	0	0	0	160 € (16 nights)	100 € (10)	§
Co-payment for hospitalization for supportive treatment, 7 nights ($p_{supportive\ treatment}$), [€]*§	23.8 € (p = 0.34)§*	0	0	0	0	§
Co-payment for rehabilitation treatment (10 nights), [€]*§	140 €§*	0	0	120 €§ (14)	140 €§ (14)	§

Co-payment for ostomy care (10€ / month)[§] [€]	0		0	0	120 €	0	§
Cost for medication and ostomy care (non-refunded)	0		0	0	120 €	0	120 €
Total co-payment if exceeding statutory threshold[§]	-		-	-	-	-	411 €[§]
Loss of salary (weeks), [€][$]	All patients	3356.84 € (36 weeks)	139.87 € (1.5 weeks)	93.25 € (1 week)	466.23 € (5 weeks)	466.23 € (5 weeks)	4314.6 € (not working)
	Working subgroup	11903.69€ (36 weeks)	495.99 € (1.5 weeks)	330.66 € (1 week)	1653.29 € (5 weeks)	1653.29 € (5 weeks)	15300 € (not working)
	Retired subgroup	0€ (0 weeks)	0€ (0 weeks)	0€ (0 weeks)	0 €	0 €	0 €
Total costs per year [€]	All patients	**3692.20 €**	**202.99 €**	**135.33 €**	**1058.31 €**	**778.31 €**	**4866.25 €**
	Working subgroup	**12239.05 €**	**63.12 €**	**42.08 €**	**2245.37€**	**1965.37 €**	**15851.65 €**
	Retired subgroup	**335.36 €**	**559.11 €**	**372.74 €**	**592.08 €**	**592.08 €**	**551.65 €**

Table S2: Overview of costs per state for resection after chemoradiotherapy (CRT, II). Relevant data for income calculation was retrieved from Table 3 and Table 4. § Co-payment expenses were cropped at the general limit of 822 € / month (primary treatment) or 411 € /month (recurrent disease). Two one-way drives were calculated per treatment. Follow-up examination schedule was time-dependent according to current guidelines (see main article). Imaging visits during follow-up might be combined so that the number of drives does not total the number of examinations. Driving expenses were calculated from patient-relevant co-payment and costs for non-refunded drives. *Maximum co-payment for hospitalization: 280 € / year (28 nights). $ Loss of salary was calculated from the difference between gross income and the general limit for sickness pay (70% of gross income) after 6 weeks of absence from work. In case of combined failure, no resumption of work was assumed and the difference between average income and disability pension was calculated according to Table 4. † No total number calculated – ongoing systemic / palliative treatment after diagnosis of recurrence, no co-payment necessary (exceeding co-payment threshold).

**CRT + resection (Group II)**	**Transition state**	**Stable disease**		**Recurrence**		
**Costs per year**	**Primary treatment**	**Follow-up Year 1-2**	**Follow-up Year 3 - 5**	**Local**	**Distant**	**Local and distant**
Total number of drives for treatments<br>*Correlating treatment and number of drives (one-way)*	100<br>Visits for primary diagnosis: 4<br>Treatment preparation: 4<br>Radiotherapy: 56<br>Preoperative visits / staging: 4<br>Consolidative chemotherapy: 32	6<br>2x Rectoscopy + clinical examination, 1x CT chest / abdomen: 2	4<br>1x Rectoscopy + clinical examination, 1x CT chest / abdomen: 2	6<br>Visits for diagnostics / follow-up: 4<br>Resection: 2	6<br>Visits for diagnostics / follow-up: 4<br>Resection: 2	†<br>Visits for diagnostics / staging: 4
Co-payment for transportation [€]§<br>*Correlating treatment and number of drives (one-way)*	40 € (8)<br>Visits for primary diagnosis: 2<br>Radiotherapy: 2<br>Resection: 2<br>Adjuvant chemotherapy: 2	0	0	10 € (2)<br>Resection: 2	10 € (2)<br>Resection: 2	n.a. §
Transportation costs, non-refunded [€]<br>*Correlating treatment and number of drives (one-way)*	42.08 € (8)<br>Visits for primary diagnosis: 2<br>Treatment preparation: 2<br>Preoperative visit: 2<br>Staging: 2	31.56 (6)<br>Follow-up visits (see above)	21.04 (4)<br>Follow-up visits (see above)	21.04 €<br>Visits for diagnostics / staging: 2<br>Follow-up visits: 2	21.04 €<br>Visits for diagnostics / staging: 2<br>Follow-up visits: 2	21.04 € (4)<br>Visits for diagnostics / staging: 4
Co-payment for medication [€], number of prescriptions§	120 € (12)	0	0	20 € (2)	20 € (2)	§
Co-payment for hospitalization for resection (number of nights)*§	160€ (16)	0	0	160 € (16 nights)	100 € (10)	§
Co-payment for hospitalization for supportive treatment, 7 nights ($p_{\text{supportive treatment}}$), [€]*§	16.10 € (p = 0.23)§*	0	0	0	0	§
Co-payment for rehabilitation treatment (14 nights), [€]*§	120 € (14)§*	0	0	120 €§ (14)	140 €§ (14)	§

Co-payment for ostomy care (10€ / month)§ [€]	120 €		120 €	120 €	120 €	120 €	§
Cost for medication and ostomy care (non-refunded)	120 €		120 €	120 €	120 €	120 €	120 €
Total co-payment if exceeding statutory threshold§	-		-	-	-	-	411 €§
Loss of salary (weeks), [€]$	All patients	3823.07 € (41 weeks)	93.25 € (1 week)	93.25 € (1 week)	466.23 € (5 weeks)	466.23 € (5 weeks)	4314.6 € (not working)
	Working subgroup	13556.98 € (41 weeks)	330.66 € (1 weeks)	330.66 € (1 week)	1653.29 € (5 weeks)	1653.29 € (5 weeks)	15300 € (not working)
	Retired subgroup	0€ (0 weeks)	0€ (0 weeks)	0€ (0 weeks)	0 €	0 €	0 €
**Total costs per year [€]**	All patients	**4545.15 €**	**364.81 €**	**354.29 €**	**1037.27 €**	**997.27 €**	**4866.25 €**
	Working subgroup	**14279.06 €**	**271.56 €**	**261.04 €**	**2224.30€**	**2184.33 €**	**15851.65 €**
	Retired subgroup	**772.08 €**	**602.22 €**	**591.70 €**	**571.04 €**	**531.04€**	**551.65 €**

Table S3: Calculated transition probabilities per cycle for single transition states. Total probabilities per cycle may not total 1 due to rounding errors.

	Cumulative probability *p* per transition state					
	**Non-operative Management (NOM, I)**					
Cycle (Year)	Stable disease	Local recurrence	Distant recurrence	Local + distant recurrence	Stable after resection for local recurrence	Death
1	0.813	0.160	0.010	0	0	0.017
2	0.736	0.043	0.029	0.007	0.149	0.035
3	0.699	0.016	0.032	0.011	0.166	0.075
4	0.679	0.008	0.026	0.014	0.156	0.117
5	0.658	0.008	0.023	0.015	0.139	0.157
	**CRT + resection (II)**					
Cycle (Year)	Stable disease	Local recurrence	Distant recurrence	Local + distant recurrence	Stable after resection	Death
1	0.858	0.019	0.067	0	0	0.056
2	0.784	0.012	0.088	0.006	0.022	0.088
3	0.743	0.006	0.073	0.013	0.039	0.127
4	0.705	0.005	0.063	0.016	0.048	0.164
5	0.672	0.008	0.050	0.017	0.053	0.200

Figure S2 + S3: Specification of costs per treatment option and transition state (total costs for treatment and 5 years follow-up, [€]). A2: Costs for Non-operative Management (NOM, I), A3: Costs for chemoradiotherapy (CRT) followed by resection (II). Costs for “Death” were set at 0 € (absorbing state).

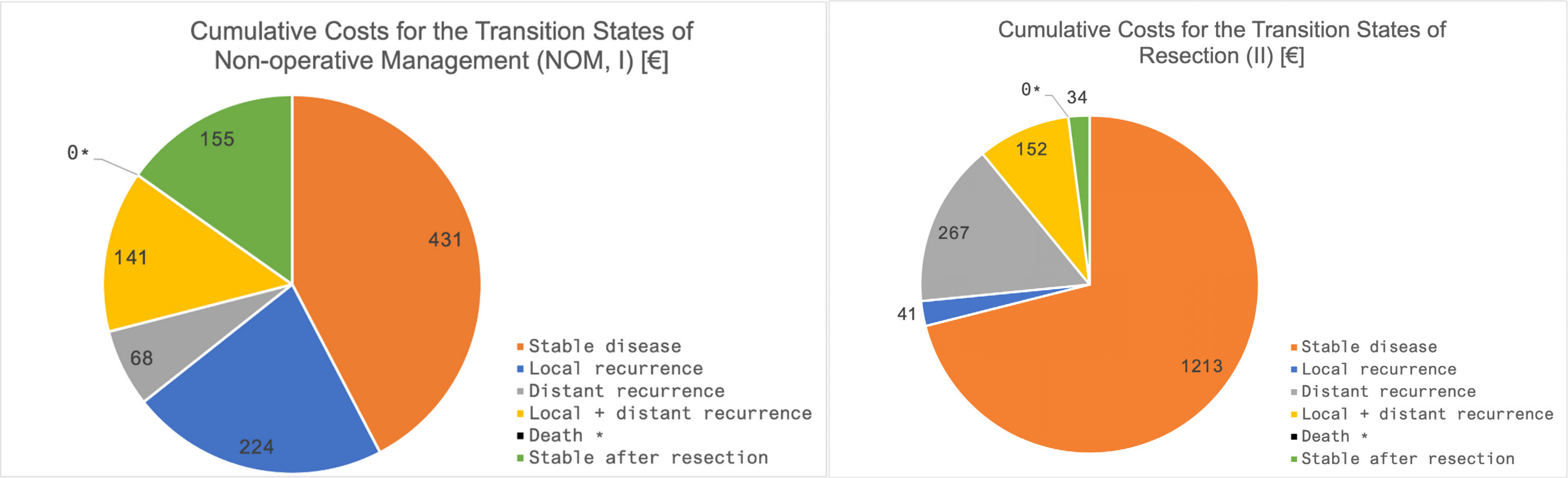

Supplement: Supplementary file 1 [file cancers-16-01281-s001.zip › cancers-2935680-SI.pdf]
